# Factors associated with single-room assignment among patients admitted through the emergency department during influenza epidemics

**DOI:** 10.1371/journal.pone.0237214

**Published:** 2020-08-07

**Authors:** Donia Bouzid, Benoit Visseaux, Anne Perozziello, Xavier Lescure, Xavier Duval, Enrique Casalino, Jean Christophe Lucet, on behalf of the ED influenza management study group

**Affiliations:** 1 IAME, INSERM, Université de Paris, Paris, France; 2 Emergency Department, AP-HP, University Hospital Bichat-Claude Bernard, Paris, France; 3 AP-HP, University Hospital Bichat-Claude Bernard, Virology, Paris, France; 4 Infectious Disease Department, AP-HP, University Hospital Bichat-Claude Bernard, Paris, France; 5 Clinical Investigation Center, AP-HP, University Hospital Bichat-Claude Bernard, Paris, France; 6 Infection Control Unit, AP-HP, University Hospital Bichat-Claude, Paris, France; University of New South Wales, AUSTRALIA

## Abstract

**Introduction:**

Every winter, emergency departments (EDs) face overcrowding with patients presenting influenza-like symptoms, and organisational issues such as single room assignment and droplet precautions to avoid hospital-acquired influenza. Our main objective was to assess the impact of PCR results and patient’s severity on single room assignment.

**Methods:**

All patients admitted to Bichat-Claude Bernard Hospital through the ED and tested for influenza by PCR (GenXpert, Cepheid) or (FilmArray, BioMérieux) on a nasopharyngeal swab were retrospectively included during three influenza seasons (2015–2018.

**Results:**

Of 1,330 included patients, 278 (20.9%) had a positive PCR for influenza. The median time to obtain a PCR result was 19 hours, and 238 (18.3%) patients were assigned a single room. Among patients with positive and negative influenza PCR, 22.3% and 16.7% were assigned a single room (p = 0.03). The multivariable analysis was performed on the two first epidemic periods, excluding the third epidemic because of the concomitant use of influenza immune-chromatic test. Only level 1 of the Emergency severity index (ESI) (aOR, 1.9; 95% CI, 1.3–2.8; p < 0.01) was associated with single-room assignment. PCR result was not statistically associated with the decision of single room assignment (aOR, 1.4; 95%CI, 1.0–1.4; p = 0.07).

**Conclusion:**

A PCR positive for influenza was not significantly associated with single-room assignment. Less than one quarter of influenza patients were adequately assigned a single room, likely due to the long turnaround time of PCR result and other conflicting indications for single room-assignment. Accelerating biological diagnosis could improve single-room assignment.

## Introduction

Influenza is a huge public health problem causing 650 000 deaths every year worldwide [1]. Annual winter epidemics of influenza also cause a large number of hospitalization, about 140,000 to 960,000 each year in the US [2], that challenge the hospital management. Though most people recover within few days, influenza can cause various and serious respiratory, cardiac or neurological complications among the elderly or other fragile patients.

During each annual winter epidemic, emergency departments (EDs) face overcrowding, with patients presenting influenza-like illness (ILI) or suspicion of pneumonia. Thus, EDs must deal with organisational issues related to patients’ isolation and the risk of viral nosocomial transmission in the context of limited availability of single rooms. A study assessing the relationship between admission to double-occupancy rooms and hospital-acquired influenza showed a higher risk of acquiring influenza among patients admitted in a double-occupancy room compared to those placed in a single room [3]. In another study, the same team estimated that the hospital-acquired influenza risk was largely increased when the roommate presented an ILI [4]. Patients’ isolation with single-room assignment is the cornerstone of droplet precautions along with wearing medical masks and hand hygiene [5]. However, in most settings, single rooms are rare and not available for all patients presenting an ILI. Rapid biological confirmation is thus recommended to help with isolation and single-room assignment decisions in hospital settings.

Polymerase chain reaction testing (PCR) has the highest sensitivity compared to other diagnostic tests [6–9]. However, PCR is currently mostly performed in the laboratory and has a long turnaround time, which can delay and alter single-room assignment for patients with ILI. Point of care (PoC) PCR assays decreased the time to result, hospitalisation and mortality rates [10] but without improvement regarding the proportion of influenza-positive patients correctly assigned to single rooms.

A recent study assessing the usefulness of multiplex PCR as PoC testing in the ED showed that the percentage of patients assigned to a single room among patients positive for influenza or respiratory syncytial virus was higher, although not significantly, in those with PoC testing than in those with laboratory testing, owed to a shorter PCR turnaround time (37.1 vs 2.3 hours) [11]. As in other studies assessing PoC usefulness [10,12,13], this study did not assess for the potential others factors possibly involved in room assignment.

Our main objective was to assess the association of influenza PCR and disease severity on single-room assignment, during the first 48 hours of admission, for patients seeking the ED during influenza epidemics.

## Methods

We conducted a retrospective study at the ED of Bichat-Claude Bernard university Hospital. This is a tertiary hospital in Paris, serving a population of more than 350,000 inhabitants. The ED receives more than 80,000 visits per year. The hospital includes 455 single rooms and 276 double rooms.

All patients admitted to the hospital through the ED and tested for influenza PCR during three consecutive influenza epidemics between 2015 and 2018 were included. During the study period, two PCR were used: GenXpert^®^ (Cepheid, Sunnyvale, CA, USA) or FilmArray^®^ RP panel (BioFire^®^, BioMérieux, Marcy-L’étoile, France). All PCR assays were performed in the virology laboratory during opening hours (8 a.m. to 6 p.m. from Monday to Friday, and 8 a.m. to 1 p.m. on Saturday).

The definition of influenza epidemic periods was based on the French national surveillance system [14]. According to the national surveillance system, the first epidemic period extended from 25 January 2016 to 17 April 2016, the second from 5 December 2016 to 12 February 2017, and the third from 4 December 2017 to 17 April 2018.

Virological data were extracted from the virology laboratory database and merged with the ED database, containing clinical data, and to the medical information system, containing duration of hospital stay and room assignment. Variables included demographic characteristics, level of triage according to the Emergency Severity Index (ESI) (ranging from 1 requiring immediate, life-saving intervention without delay to 5 meaning stable, without any resources anticipated except oral or topical medications, or prescriptions) [15,16], PCR turnaround time, ward of admission, and room assignment.

The primary outcome was the single- or double-room assignment at 48 hours of hospital stay, whatever the type of unit of stay, either a clinical ward or the downstream ED unit.

Variables were summarised as frequency and proportion for categorical variables and median and interquartile range for continuous variables. Continuous variables were converted into categorical variables using their interquartile range. We compared the characteristics of the three epidemics using the Kruskal-Wallis test for quantitative variables and the χ^2^ test for the qualitative variables. Logistic regression was used to identify factors influencing the primary outcome in the multivariable analysis including: age, PCR results, weekend shifts, time between ER sampling and PCR results. During the third period, ED physicians used a point of care testing with an immune-chromatographic method in addition to PCR, therefore shifting the association between time to PCR results and room assignment. All tests were performed with a type I error of 0.05. The data management and statistical analysis were performed using Stata15^®^.

The study design has been approved by the Bichat Claude Bernard ethic committee (N°2019–029).

## Results

**Fig 1:**
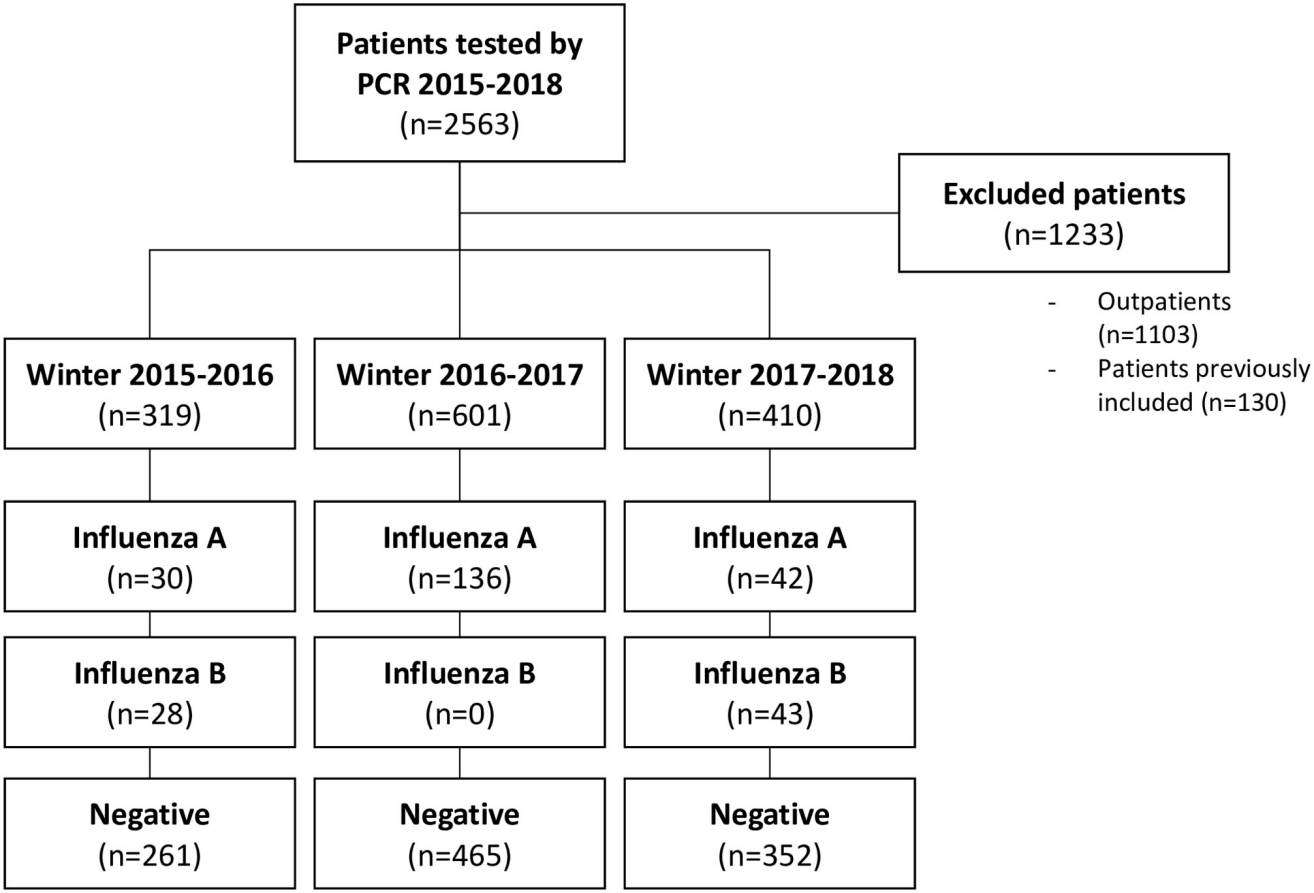
Study flowchart.

Among the 2563 patients, 1233 were not included because they were previously included in the study or not admitted after their visit at the ED. Thus, we included 1330 patients: 319, 601, and 410 during the first, second, and third epidemic period, respectively (Fig 1). A total of 278 patients (20.9%) tested positive for influenza with PCR.

The sex ratio was similar across the three periods; several differences were observed (Table 1). Patients were younger during the first period than during the second and third periods (p < 0.001); only influenza type A virus was identified during the second period, while both influenza A and B were detected during the first and third periods; the triage level distribution was also significantly different over the three periods (p = 0.02); however, the proportion of level-1 triage, representing the 221 most severe patients (16.6%) with either hemodynamic or respiratory failure was not statistically significant among the three periods (p = 0.49). Finally, the distribution of ward admission was significantly different between, with more patients admitted to a surgical ward during the second period as compared to the first and third periods. Overall, 36 (2.7%) patients were admitted to the intensive care unit (ICU) from the ED.

**Table 1:** Characteristics of included patients with ILI, during the three winter epidemics (2015–2018) at Bichat Claude Bernard Hospital.

Characteristics (n, %)	Winter 2015–2016 (319 patients)	Winter 2016–2017 (601 patients)	Winter 2017–2018 (410 patients)	p value
	(n,%)	(n,%)	(n,%)	
Gender				0.76
Female (n = 629)	147 (46.1)	297(49.41)	185 (45.2)	
Male (n = 701)	172 (53.9)	304 (50.6)	225 (54.8)	
Age (years, median, IQR)	73.9 (61.2–82.6)	79.6 (66.6–87.7)	77.4 (64.2–87.9)	<0.001
< 65 (n = 346)	96 (30.1)	139 (23.1)	111 (27.1)	
65–75 (n = 259)	73 (22.8)	110 (18.3)	76 (18.5)	
76–85 (n = 310)	82 (25.7)	136 (22.6)	92 (15.3)	
> 85 (n = 415)	68 (21.3)	216 (35.9)	131 (31.6)	
Triage level:				0.01
Level 1 (n = 221)	50 (15.7)	99 (16.5)	72 (17.5)	
Level 2 (n = 377)	104 (32.6)	147 (24.5)	126 (30.7)	
Level 3-4-5 (n = 732)	165 (51.7)	355 (59.1)	212 (51.7)	
Ward admission				<0.001
Medical (n = 942)	236 (73.9)	393 (65.4)	313 (76.3)	
Surgical (n = 156)	22 (6.9)	93 (15.4)	41 (10)	
ICU (n = 36)	11 (3.4)	13 (2.1)	12 (2.9)	
Transferred (n = 196)	50 (15.7)	102 (16.9)	44 (10.7)	

Abbreviations: PCR, Polymerase Chain Reaction; IQR, Inter Quartile Range.

The median turnaround times between ED sampling and PCR result were 19.3, 19.2, and 15.7 hours during the first, second, and third period, respectively (Table 2). In total, 238 (17.8%) patients were assigned to a single room within the first 48 hours after admission. Among those 238 patients with IL assigned a single room, 62 (26.1%) had a positive PCR.

**Table 2:** Summary of PCR results and room assignment for patients with ILI during the three winter epidemics (2015–2018), at Bichat Claude Bernard Hospital.

Characteristics (n, %)	Winter 2015–2016 (n = 319)	Winter 2016–2017 (n = 601)	Winter 2017–2018 (n = 410)	p value
Positive PCR (n = 278)	58 (18.2)	136 (22.6)	85 (21.2)	
Influenza A (n = 208)	30 (51.7)	136 (100)	42 (49.4)	<0.001
Influenza B (n = 71)	28 (48.27)	0 (0)	43 (50.5)	
Time between sampling and PCR result (hours, median, interquartile range)	19.3 (7.2–36.6)	19.2 (9.7–29)	15.6 (4.8–27.4)	
≤ 6 h	71 (22.2%)	110 (18.3)	134 (32.7)	<0.001
> 6h	248 (77.8%)	491 (81.7)	476 (67.3)	
Room assignment				
Single room (n = 238)	82 (25.7)	141 (23.5)	15 (3.6)	<0.001
Double room (n = 1092)	237 (74.3)	460 (76.5)	395 (96.4)	

Abbreviations: PCR, Polymerase Chain Reaction; IQR, Inter Quartile Range.

During the third epidemic, ED physicians had access to an influenza point of care test, which made this epidemic not comparable to the other epidemic. Indeed we observed an interaction between this third period and PCR turnaround time, therefore we have chosen to exclude the third epidemic from the logistic regression analysis. There was no association between single-room assignment and positive PCR result (Table 3). The only factors associated with single-room assignment in the univariable analysis were the level-1 triage and weekend visits to the ED. We did not observe statistically significant difference between day or night visits and the type of room assignment.

**Table 3:** Univariable and multivariable analysis of factors associated with a single room assignment for patients with ILI during two winter epidemics (2015–2017) at Bichat Claude Bernard hospital.

Factors (n, %)	Room assignment		Univariable analysis		Multivariable analysis	
	Single (n = 223)	Double (n = 697)	RR (95%CI)	p value	OR (95%CI)	p value
Age (years)*				0.21		
≤ 75 (*n = 418*)	93 (41.7%)	324 (46.5%)	1			
> 75 (*n = 502*)	130 (58.3%)	373 (53.5%)	1.2 (0.9–1.4)			
Male gender *(n = 476*)	117 (52.5%)	359 (51.5%)	1.0 (0.8–1.3)	0.82		
PCR*						
Negative (*n = 1052*)	167(74.8%)	558 (80.0%)	1		1	
Positive (*n = 278*)	56 (25.2%)	139 (20.0%)	1.3 (0.9–1.6)	0.08	1.4 (1.0–1.9)	0.07
Triage level*						
Level 2-3-4-5 (*n = 771*)	170 (76.3%)	601 (86.3%)	1		1	
Level 1 (*n = 149*)	53 (23.7%)	96 (13.7%)	1.61 (1.2–2.1)	<0.01	1.9 (1.3–2.8)	<0.01
ER sampling to PCR results*						
≤6h (*n = 181*)	35 (15.7%)	146 (20.9%)	1			
> 6h (*n = 739*)	188 (84.3%)	551 (79.1%)	1.3 (0.9–1.8)	0.09		
Weekends* (*n = 329*)	66 (29.6%)	162 (23.2%)	1.3 (1.1–1.6)	0.04		
Type of shift						
Day (*n = 647*)	155 (69.5%)	492 (70.6%	1			
Night (*n = 273*)	68 (30.5%)	)205 (29.4%)	1.1 (0.9–1.1)	0.70		

Abbreviations: PCR, Polymerase Chain Reaction.

* Included in multivariable analysis.

In the multivariable analysis. Only level 1 of triage according to ESI was associated to single-room assignment (adjusted OR,1.9; 95%CI, 1.3–2.8, p<0.01), while a positive PCR result for influenza was not (aOR, 1.46; 95% CI, 1.0–1.9; p = 0.07); turnaround time and weekends were no longer statistically associated to single-room assignment.

## Discussion

In this 3-year observational study in a large teaching hospital, we showed that patients’ severity based on ESI level 1 was the only factor associated with single room. Only 25.2% of admitted patients presenting a positive PCR for influenza were assigned to a single room. These results underline that patient’s isolation remains insufficient, despite a large number of 455 single rooms in our hospital.

Previous studies have shown that single-room assignment is efficient in reducing nosocomial infections, specifically influenza [17,18], but the availability of single rooms is often still beyond the needs during influenza seasons [19]. Despite the fact that newly built hospitals may tend to include larger numbers of single rooms [20], the placement of patients with high-risk respiratory viral infection, notably influenza infection, remains a challenge. In addition, the proportion of influenza infections is usually low among patients presenting an ILI during outbreak periods, about 20% in our study and in previous works [21,22]. Thus, a rapid virological diagnosis is required to appropriately decide which patient with ILI should be assigned to an available single room. However, we did not find an association between the time to result, defined as the delay between ED sampling and PCR results, and single-room assignment. This may be largely explained by a minimal time to result in our study always being longer than 3 hours due to sample transportation to the laboratory and PCR delay. Due to these delays and the limited opening hours of the laboratory, the median time to result was 19 hours. This might remain too long to allow the consideration of the result by the ED physician at the decision-making time. This is in line with a recent randomised study that showed that performing influenza PoC PCR improved the proportion of patients isolated with a confirmed respiratory virus infection (17 vs 9%, p = 0.02) and reduced the hospital length of stay [11]. Furthermore, in a post-hoc analysis, the same authors showed that a PCR result obtained in less than 2 hours reduced the duration of antibiotic treatment by half and reduced the length of hospital stay by about 3 days [23]. Further studies are required to evaluate the impact of PoC influenza PCR and placement in single rooms.

In the current work, single-room assignment was associated with patient severity, defined as level-1 triage of the ESI. It has been shown that severe patients have a longer length of stay in the ED [24], but no previous study has assessed the impact of the ESI on single-room assignment in the downstream unit.

To our knowledge, our study is the first to assess factors leading to single-room assignment in adult patients presenting an ILI. Due to the study’s retrospective and observational design, our results reflect the actual practice in a French ED and contrast with a recent study [11]. As any patient presenting with ILI is required to be placed in a single room pending PCR result, our low proportion of influenza-positive patients in single room was surprising and highlight the difficulties of such organization in the emergency context.

Our work presents several limitations. Firstly, it was a retrospective and monocentric study. During the study period, several changes occurred in the global management of patients presenting an ILI, especially during the third epidemic period: a new multiplex PCR was available and allowed faster results, with a median turnaround time of 15.6 vs 19.3 hours for the previous epidemics. In addition, a new algorithm for the influenza diagnosis based on an immuno-chromatographic rapid influenza diagnostic test (RIDT) was applied in the ED at the same period, in parallel to the PCR testing at the virology laboratory. Based on this algorithm, all patients presenting an ILI were initially tested using a PoC RIDT; only patients admitted in the hospital were tested by a PCR in the virology laboratory. The observed poor accuracy of the RIDT might explain the lower frequency of single-room assignment of patients with a positive PCR. Excluding the third epidemic period, the proportion of patients with positive PCR assigned a single room was higher, at 24%. This distinct period also illustrates that single room assignment for influenza-positive patients remains a year to year challenge that could not be resolved by a rapid but less reliable PoC method. Secondly, we could not retrieve the detailed occupancy rates during the epidemics, and the actual availability of single rooms remained unknown. This parameter should, intuitively, also be linked to single-room assignment. We also did not know if some influenza positive patients may have been rarely cohorted in double rooms. Finally, the ED database lacked information for two critical time points: the time of decision for hospital admission and the time of actual information of the ED physician of the PCR result. The proportion of patients with a positive influenza PCR assigned to a single room remained low over the three periods. However, in a 2018–2019 pilot study conducted in our ED with a Multiplex PCR point of care method, allowing a result within one hour, 80% of patients were assigned to a single room [25]. Taken together with the result of the current study, this suggests that PCR turnaround time should be greatly shortened for decision making of room assignment.

## Conclusion

Hospital-acquired influenza is an old and well-known threat that the physicians must tackle by applying preventive measures based on accurate and rapid diagnosis. The optimal protocol to optimise both costs and patient isolation management is yet to be precisely identified. Our results highlighted that a large majority of influenza-positive patients were still assigned double-occupancy rooms and it is likely due to the long turnaround time of PCR results. As most ED physician decisions are made in a data frame of a few hours, shortening the time to result by implementing point of care PCR should improve patients’ management, facilitate the ED organisation, and improve isolation and droplet precautions during the overcrowded influenza season.

## Supporting information

S1 File (DOCX)

## Abbreviations

D: Emergency department
ESI: Emergency severity index
ICU: Intensive care unit
ILI: influenza like illness
PCR: Polymerase Chain Reaction
POCT: Point of care test
RIDT: Rapid influenza diagnostic test
