# Supplementary material for: Factors associated with single-room assignment among patients admitted through the emergency department during influenza epidemics
Source: PLoS One. 2020 Aug 7;15(8):e0237214. doi: 10.1371/journal.pone.0237214 (PMC7413401; doi:10.1371/journal.pone.0237214)
Supplement: S1 File — (DOCX) [file pone.0237214.s001.docx]

| Level | Name | Description | Examples |
| --- | --- | --- | --- |
| 1 | Resuscitation | Immediate, life-saving intervention required without delay | Cardiac arrest  Massive bleeding |
| 2 | Emergent | High risk of decoration, or signs of a time-critical problem | Chest pain  Asthma attack |
| 3 | Urgent | Stable, with multiple types of resources needed to investigate or treat (such as lab tests plus X-ray imaging) | Abdominal pain  High fever with cough |
| 4 | Less urgent | Stable, with only one type of resource anticipated (such only an X-ray, or only sutures) | Simple laceration  Pain on urination |
| 5 | Nonurgent | Stable, with no resources anticipated except oral or topical medication, or prescription | Rash  Prescription refill |

ESI Triage levels according to ESI.

[1] Emergency Severity Index (ESI): A Triage Tool for Emergency Department Care, Version 4 n.d.:114.
